# Characterizing air and noise pollution and their determinants in elementary schools in Accra, Ghana

**DOI:** 10.1088/2752-5309/ae27eb

**Published:** 2025-12-15

**Authors:** Carissa L Lange, Sierra N Clark, Abosede S Alli, James Nimo, Kate A Kyeremateng, Samuel Agyei-Mensah, Youssef Oulhote, Allison F Hughes, Majid Ezzati, Raphael E Arku

**Affiliations:** 1 Department of Environmental Health Sciences, University of Massachusetts, Amherst, MA, United States of America; 2 School of Health and Medical Sciences, City St George’s, University of London, London, United Kingdom; 3 Department of Physics, University of Ghana, Legon, Accra, Ghana; 4 Department of Environmental & Sustainable Engineering, State University of New York, Albany, NY, United States of America; 5 Department of Biostatistics and Epidemiology, University of Massachusetts, Amherst, MA, United States of America; 6 Department of Geography and Resource Development, University of Ghana, Accra, Ghana; 7 Department of Environmental Medicine and Climate Sciences, Icahn School of Medicine at Mount Sinai, New York, NY, United States of America; 8 Department of Epidemiology and Biostatistics, School of Public Health, Imperial College London, London, United Kingdom; 9 Regional Institute for Population Studies, University of Ghana, Accra, Ghana; 10 Abdul Latif Jameel Institute for Disease and Emergency Analytics, Imperial College London, London, United Kingdom; 11 MRC Centre for Environment and Health, School of Public Health, Imperial College London, London, United Kingdom

**Keywords:** air pollution, environmental noise, noise annoyance, children, elementary schools, Accra, Ghana

## Abstract

In Sub-Saharan African (SSA) cities, elementary school environments may significantly contribute to children’s exposure to environmental pollution, potentially affecting their health, development, and learning. Despite children spending much of their day at school, limited data exists regarding levels, inequalities, and determinants of air and noise pollution in school settings, particularly in rapidly urbanizing regions. As part of the Accra School Health and Environment Study (ASHES), we assessed air and noise pollution in primary schools across the Greater Accra Metropolitan Area, one of SSA’s fastest-growing metropolises, and explored determinants of pollution levels around these schools. We conducted weeklong measurements of fine particulate matter (PM_2.5_), black carbon (BC), and sound pressure levels in 90 schoolyards (74% public, 26% private). We assessed schoolyard characteristics (surface type, greenness, road proximity) and examined their associations with pollutants using generalized additive models. Additionally, we evaluated 1037 child responses to noise annoyance surveys. Annual equivalent PM_2.5_ concentrations exceeded WHO guidelines by 2–13 times (11–65 *µ*g m^−3^). Median noise levels (57 dBA) surpassed Ghana EPA standards at >60% of schools, coinciding with 60% of students reporting high noise annoyance. BC and noise were higher in public and more urban schools. In the most urbanized district, all pollutants were inversely associated with neighborhood socioeconomic status. Lower greenness correlated with higher BC levels; associations with other spatial factors were weak or not statistically significant. These findings underscore the need to reduce air and noise pollution at urban SSA schools and promote healthier, quieter environments that support learning and development.

## 1. Introduction

Like all urban residents, children living in growing sub-Saharan African (SSA) cities benefit from the opportunities urban living offers. For example, urban children have health, developmental, and educational advantages over their rural counterparts [1–3]. However, living in cities also presents unique challenges, including exposure to poor environmental conditions such as air and noise pollution. Ambient air pollution has become a major public health concern in SSA cities as consequence of the ongoing urban and economic growth in the region [4, 5]. Environmental noise may also be a concern but has largely been understudied. Although times spent during daily commute are equally important, children’s exposure to air and noise pollution occurs primarily at home and school environments, the two most important places where they spend much of their time. A safe, clean, and quiet school environment may foster physical, cognitive, and social development of children, while promoting general well-being and offering a positive space to learn and play [6]. Yet, little is known about the levels, variations, and determinants of air and noise pollution in schools in rapidly urbanizing SSA cities.

Data from studies, primarily conducted in cities across Western countries with distinct emission profiles, have linked childhood exposure to fine particulate matter (PM_2.5_) to adverse lung and respiratory, cardiovascular, and metabolic outcomes [7–11]. Additionally, children’s exposures to air pollution and transportation noise have been shown to influence sleep, behavior, learning, and cognition [12–20]. Consequently, studies have linked these pollutants to reduced academic performance (e.g. test scores, grade point averages, inattention) and increased school absenteeism, both of which have potential implications for future educational attainment and socioeconomic status (SES) in adulthood [19–25].

However, due to the complexity of SSA’s urban environments, air pollutant emissions and environmental noise have both shared (e.g. road traffic) and distinct (e.g. loud street music from small businesses, household biomass use) local sources. Additionally, exposure patterns and school locations in SSA cities differ from those in high-income countries [26, 27]. A previous study in Accra, Ghana, found that schoolchildren’s personal PM_2.5_ exposures were more than double those observed in the US, and factors such as schools’ proximity to major roads, community biomass uses near schools, and materials of school ground surfaces all influenced children’s personal exposures [28]. Still, it is unknown how much consideration is given to air quality and environmental noise in urban schools in SSA and how much data informs the locations of schools to ensure sufficiently clean and quiet environments conducive to healthy childhood development. Measurement data can motivate policies that specifically address air and noise pollution in existing schools or as new schools are being built, given the ongoing urban population growth and push for universal education in the region.

This paper provides a comprehensive assessment of school-related exposures including air pollution, noise, and green space within the urban environment in a major SSA city. We examined PM_2.5_, black carbon (BC), and environmental noise pollution in both public and private elementary schools across the Greater Accra Metropolitan Area (GAMA), the most urbanized area of Ghana. We also investigated discordance/concordance, and socioeconomic inequalities, and quantified potential determinants of exposures. This study was part of the Accra School Health and Environment Study (ASHES), which was designed to examine the impact of multiple environmental exposures on key childhood health and developmental outcomes in a major SSA city. To our knowledge, this is the first study to provide a comprehensive assessment of outdoor air and noise pollution across elementary schools in one of SSA’s fastest growing metropolises. By characterizing environmental exposure data across a diversity of school settings, our findings aim to inform public health and school-level interventions and urban planning efforts in similar low-resource urban environments.

## 2. Methods

### 2.1. Ethical approval

The study was approved by the Institutional Review Boards of the University of Massachusetts Amherst [00003909] and University of Ghana, Legon [ECH 149/ 18–19]. Additional permission to conduct research in schools was secured at the national, regional, and district offices of the Ghana Education Service (GES), along with approval from school head teachers. Written informed consent was obtained from school administrators and parent/guardian(s) as well as assent from the children. The overall research was conducted in accordance with the principles embodied in the Declaration of Helsinki and followed the Ghanaian local statutory requirements.

### 2.2. Study setting and design

**Figure 1.**
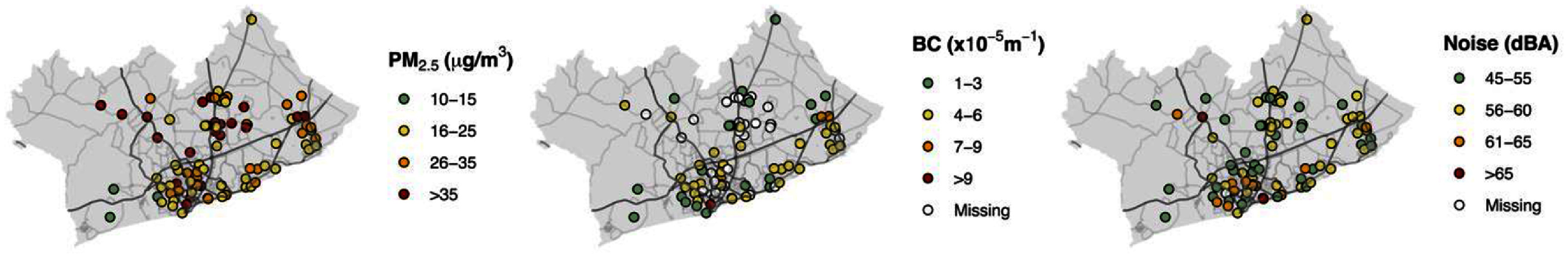
Map of the GAMA and schools included in ASHES. The colors indicate each school’s associated annual equivalent PM_2.5_ concentrations, BC absorbance, or noise levels.

Details of the ASHES design, school selection, and data are described elsewhere [29]. Briefly, our study was conducted in the GAMA, the largest metropolis in Ghana (∼1500 km^2^) and home to nearly 6 million residents [30]. Ghana operates a 6-3-3-4 school system (i.e. from elementary to university). Basic education is compulsory and offered by both public and private schools. Public schools are free but are often considered overcrowded and lack resources, whereas private options are fee-paying and often favored by higher income earners for their purported better education. ASHES selected 100 schools from a geocoded list of ∼700 public and ∼2000 private primary schools obtained from GES for participation, but ten declined, leaving 90 public (74%) and private (26%) primary schools (figure 1). The schools were from all 13 GAMA districts. Low-income communities and localities in and around the most urbanized Accra (AMA) and Tema (TMA) metropolises have more elementary schools than the other adjoining districts in the GAMA. In these districts, we selected eight or nine public schools and two or three private schools per district, as opposed to four public and one private school in peri-urban districts. Public schools were purposefully over-represented, as that is where many national and local policy interventions are implemented. We recruited 1037 schoolchildren (mainly in grades 4 and 5) aged 8–12 years from the 90 participating schools for assessment of multiple health indicators and outcomes. Specifically, we targeted a total of ten 4th graders from each school with the aim of ensuring equal representation of both genders, and if this target was not met, 5th graders were invited to participate to complete the sample. This age group was chosen in part because they were just old enough to accurately respond to survey questions.

Between June 2022 and May 2023, we conducted week-long (7 d) measurements of ambient PM_2.5_, BC, and sound pressure levels in the school yards. The monitors were deployed in five schools in each measurement week (e.g. Monday–Monday schedule) during the school months. Trained field assistants completed a structured survey during the deployment to gather information on the built environment of each school, including details of the surfaces of the schoolyard and playground (grass, paved, packed and loose dirt), ventilation (window types), neighborhood type (residential, commercial/business/industrial, background/other), greenness/vegetation, and commercial activity near the school (none, markets, other) (table S4).

We focused our measurements on outdoor schoolyard environments because most schools in Accra rely on natural ventilation, leading to minimal differences in air pollution levels between outdoor and in-classroom settings. In our study, 96% (*n* = 86) of the schools had louver blades, wooden, or hollow block windows that supported natural ventilation; only 4% had glass windows which could support mechanical ventilation, but they also relied on natural ventilation. Our decision to focus on schoolyards was also guided by prior studies in Accra neighborhoods and homes, as well as personal exposure assessments of schoolchildren, which found minimal differences in PM_2.5_ and ultrafine particle levels between indoor and outdoor environments in the absence of direct household biomass burning [28, 31, 32]. Specifically for schoolchildren, factors such as proximity of schools to major roads, materials of schoolyard surfaces, and biomass use in the surrounding areas are known determinants of their personal PM_2.5_ exposure in Accra [28]. These findings suggest that outdoor measurements at schools are a reasonable proxy for estimating schoolchildren’s exposure in this context.

### 2.3. Data collection and analytical methods

#### 2.3.1. PM_2.5_ and BC

We measured both weeklong integrated gravimetric (filter-based) and real-time/continuous (minute-by-minute) PM_2.5_ concentrations using the ultrasonic personal aerosol sampler [33] and the ZeFan [34], respectively. PM_2.5_ mass was collected on 37 mm Teflon membrane filters and were weighed pre- and post-sampling using a MTL AH500 automated robotic scale in a climate-controlled room. Weekly integrated BC concentrations were estimated using the absorption coefficient (light absorbance) of the post-weighed PM_2.5_ filters, which were determined by applying an image-based reflectance method [35]. Filters from 28 samples were excluded from the BC analysis due to filter loss or failure of the sample to meet our pre-specified inclusion requirements that instruments had to (i) operate for ⩾75% of the measurement period (i.e. at least 5 of 7 d) and (ii) record an average flow rate within 10% of the intended rate (1 l per minute). However, for PM_2.5_, the missing integrated data were replaced with collocated corrected continuous monitor data. Specifically, we applied a correction factor (CF) to the minute-by-minute continuous PM_2.5_ data, consistent with previous studies [36, 37]. CFs were calculated at schools that had both valid integrated and continuous data, such that the average of the continuous PM_2.5_ data was set equal to the integrated sample. On average, the continuous monitors underestimated concentrations during the Harmattan season and overestimated in the non-Harmattan. Consequently, we applied season-specific mean CFs (Harmattan = 1.12; non-Harmattan = 0.83) to adjust continuous data at schools missing integrated samples.

Furthermore, since measurements did not occur simultaneously across all 90 schools to allow for school-to-school comparison, we accounted for the potential influence of time/season by applying a temporal adjustment factor (TAF). TAF was computed separately for each measurement week using data from ten fixed ambient monitoring stations located throughout the GAMA. Details regarding the ambient sites and the adjustment procedure have been previously published elsewhere [38]. In brief, a TAF for each measurement week was calculated as the ratio of the mean PM_2.5_ or BC across all fixed sites during that week to the annual mean PM_2.5_ or BC across those same sites. These weekly TAFs, ranging from 0.39 to 4.67 for PM_2.5_ due to strong temporal variability in air pollution in Accra [36, 37], were then applied to each school’s PM_2.5_ (or BC) measurement. This resulted in an annual equivalent mean concentration for each school, enabling school-to-school comparison.

In addition to the annual equivalent mean concentrations, we also derived a unique school pollution metric that reflects exposures during school hours only—defined as 7 am to 3 pm, Monday through Friday (40 h/week total), using the corrected minute-by-minute/continuous data.

#### 2.3.2. Environmental noise

Ambient sound pressure levels were measured in one-minute integrated intervals in A-weighted decibels (dBA) using Convergence Instrument’s Noise Sentry sound level meter (SLM) [39]. We collected 445-school days of sound measurements, with one school having lost a full week of data due to equipment theft. We calculated a metric (*L*_day(school)_) of equivalent continuous sound levels, integrated across measurements conducted on school days (Monday–Friday) and during typical school hours (7 am–3 pm). The *L*_day(school)_ metric excluded data during break periods (i.e. hours when children go out into the schoolyard to play, typically between 9:30 am–10 am and 12 pm–12:30 pm), as we wanted to characterize the environmental noise that children experienced rather than the sound they created themselves (i.e. from laughing/playing/talking) (35 h/week total). The hourly equivalent continuous sound levels during the break periods were on average 0.7 dBA higher than the other school-time hours. Further, we also computed Leq_wk_, a measure of equivalent continuous sound levels integrated over the entire measurement week. Finally, we calculated the intermittency ratio (IR) which describes the amount of intermittent sound in a specified period [40]. The IR represents the percentage (0%–100%) of sound energy that is created from distinct sound events that surpass a fixed cut-off value above the site and day-specific average equivalent continuous sound level. For this study, we used a threshold of 3 dBA above, in line with previous studies conducted in Accra [41]. Additional details regarding the IR calculation are included in the Supplementary Information.

#### 2.3.3. Assessment of noise annoyance

The children responded to a noise annoyance survey through a set of 5-point verbal and 11-point numeric questions, in line with the 2003 Technical Standard by the International Standards Organization (ISO/TS 15666:2003) [42]. The questions evaluated the students’ self-reported annoyance to noise generated by road-traffic, aircraft/planes, industry/factories, business/stores/churches, and neighbors within the past 12 months while in school (table S3). We caution that while these survey questions have been used as a validated instrument in adult populations and household settings for decades, they have not been validated specifically in children [42]. We used the students’ responses to calculate three metrics describing the percentage of students ‘highly annoyed’ (HA) by noise exposure at school, where HA*_N_* refers to numerical values of 8, 9, and 10 from the 11-point numeric questions, and HA*_V_* considers the top two verbal response categories (i.e. very and extremely) to the 5-point verbal questions (Likert scale). The final metric, HA_VW_, represents the top two verbal response categories to the 5-point verbal questions, with ‘extremely’ weighted in full and ‘very’ weighted by a factor of 0.4. Because we observed consistency across all three HA metrics (table S1), only HA*_N_* results are reported.

#### 2.3.4. Features of the schools and neighborhood environments

We gathered information on known spatial and meteorological factors that could influence air and noise pollution in Accra school settings. We quantified vegetation greenness (range: 0–1) at each school using the normalized difference vegetation index (NDVI). NDVI values were calculated from spectral bands of green vegetation in Landsat 8 satellite imagery (U.S. Geological Survey) with the least amount of cloud cover (0.02%) using a circular buffer with a radius of 100 m [43]. NDVI values across our schools ranged 0–0.5, thus we further classified each school as either ‘bare soil’ (⩽0.1) or ‘sparse vegetation’ (0.2–0.5), with none having ‘dense green vegetation’ (0.6–0.9). We computed distance of each school to the nearest major and secondary road using OpenStreetMap road network data. The schools were further categorized as residing within or beyond 500 m of a major road [44]. Meteorological variables, including daily temperature and relative humidity were collected at a fixed peri-urban background location, while rainfall data were obtained from the Ghana Meteorological Agency.

Further, we examined the association between a measure of SES of the neighborhood containing the school and the measured air and noise pollution levels. Indices of neighborhood SES were computed as the median log equivalized household consumption (Ghanaian Cedi (GH₵)) of each census enumeration area (EA; the smallest geographic unit for census enumeration), based on household expenditures and rent information collected by the Ghana Living Standards Survey (GLSS) Round 6 [45]. The GLSS data were combined with the 2010 Ghana Population and Housing Census dataset to extrapolate relationships between consumption, area, and a variety of demographic features. We assessed school neighborhood SES on a continuous scale as well as by categorizing each school as below or above the median neighborhood SES.

#### 2.3.5. Descriptive summaries

We reported summary statistics (medians and interquartile ranges (IQRs)) for PM_2.5_, BC, *L*_day(school)_, Leq_wk_, and IR for all schools. We examined potential differences in air and noise pollutants by school type (public vs private), schoolyard surface (paved vs unpaved), and district (comparing the city core Accra Metropolitan Area (AMA), the port and industrial city of Tema (TMA), and all other adjoining peri-urban districts). We also examined the spatial correlation between the air pollutants and environmental noise to understand their degree of concordance. Further, we used Pearson correlation coefficients (*r*) to examine the associations between the pollutants and various features of the school environment including SES index, distance to nearest major road, and NDVI, with correlations interpreted as weak (<0.3), moderate (0.3–0.5), or strong (>0.5). We also specifically examined the pollutants in relation to neighborhood SES for schools residing in the most urbanized AMA. Finally, we described differences between HA*_N_* across districts and estimated a noise annoyance exposure-response curve with focus on noise generated by road-traffic at school. The HA*_N_* to road-traffic noise was calculated within 5 dB bins and the quadratic relationship was investigated.

#### 2.3.6. Determinants of school-level PM_2.5_, BC, and noise pollution

We used generalized additive models to examine the associations of the measured pollutants with their potential determinants. The models were adjusted for district (AMA, TMA, other), distance to major and secondary roads, commercial activity, NDVI score, schoolyard surface, school type (private vs public), and season (Harmattan vs non-Harmattan), using smoothing spline functions of weekly average rainfall, temperature and relative humidity, as these were found to be nonlinearly related to the pollutants. The variables were selected based on existing literature on determinants of air pollution in Accra [28, 45, 46]. The distribution of both PM_2.5_ and BC concentrations were right-skewed, and as such, were log-transformed to achieve normality. These models were used primarily for exploratory analysis.

## 3. Results

### 3.1. PM_2.5_ and BC concentrations and sound levels

**Figure 2.**
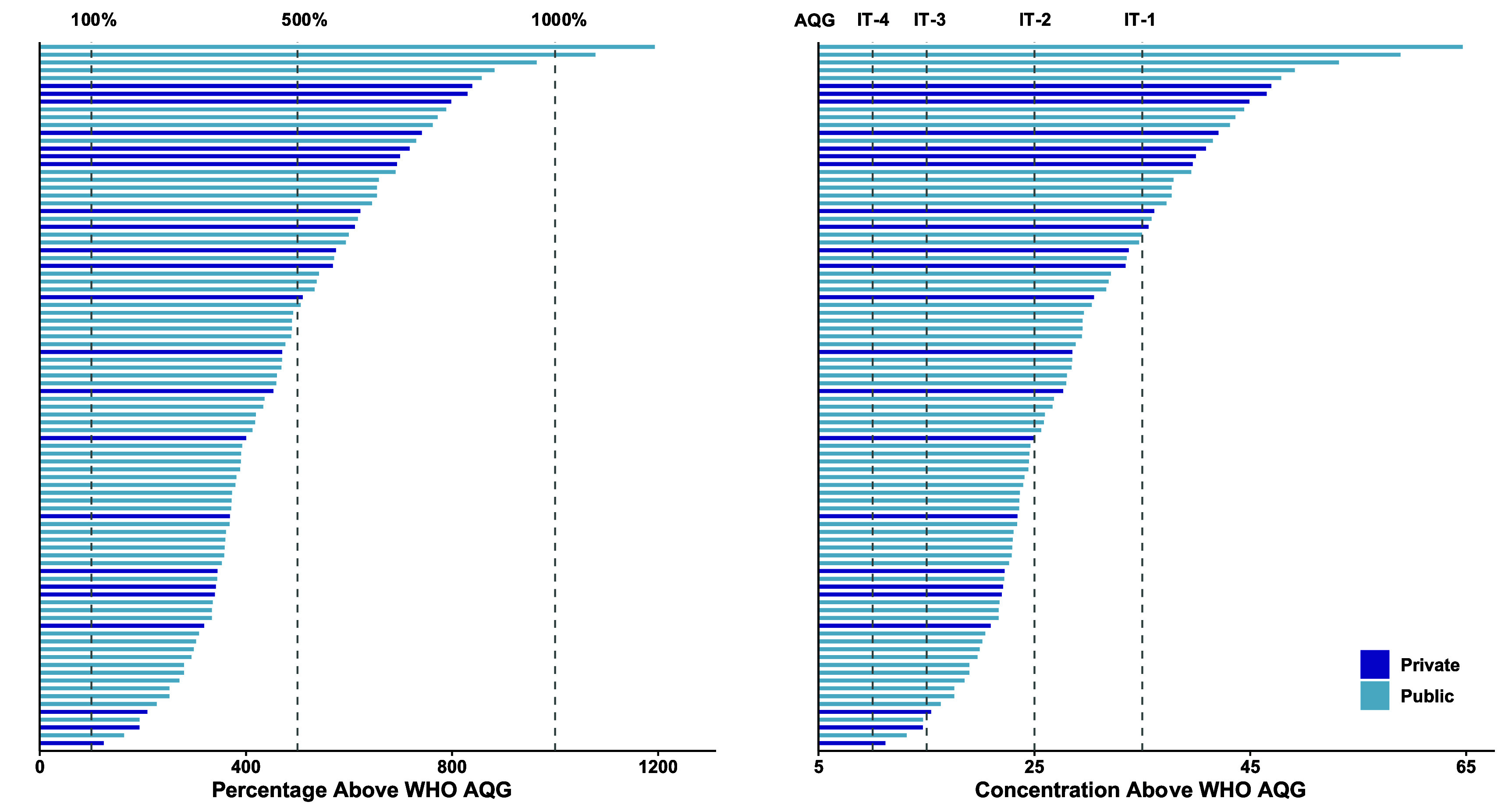
Percentage (A) and concentration (B) of annual equivalent PM_2.5_ above the WHO annual AQG of 5 *µ*g m^−3^. IT-1–IT-4 represent WHO interim targets that guide reduction efforts. Targets range from 10 (IT-4) to 35 (IT-1) *µ*g m^−3^.

**Figure 3.**
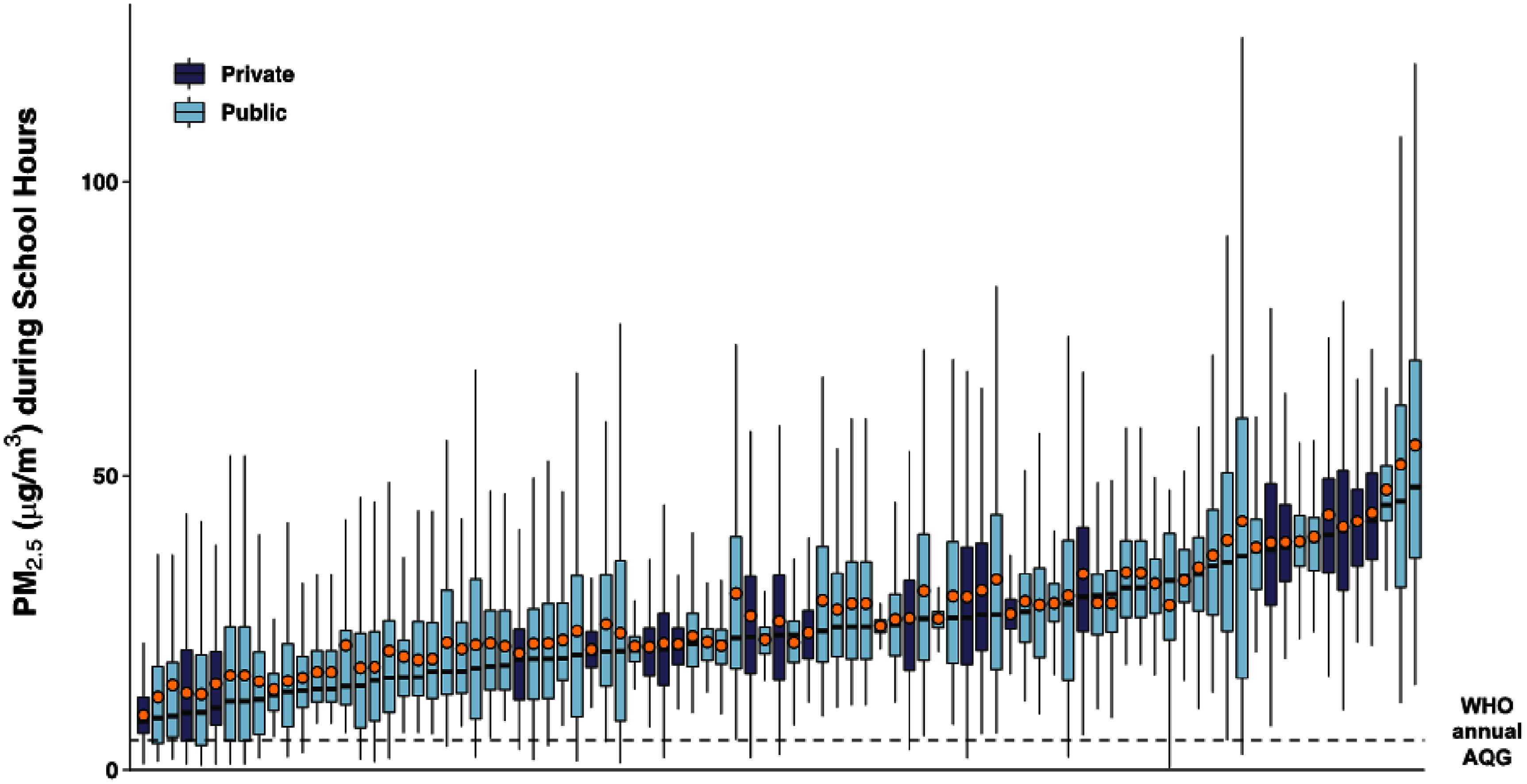
Annual equivalent PM_2.5_ concentrations during school hours (7 am–3 pm) across all schools. The box plots represent the interquartile range (IQR), with the horizontal line inside each box indicating the median, and the orange points representing the mean concentrations at each school. The dashed line indicates the WHO’s annual air quality guideline (AQG) of 5 *µ*g m^−3^.

**Figure 4.**
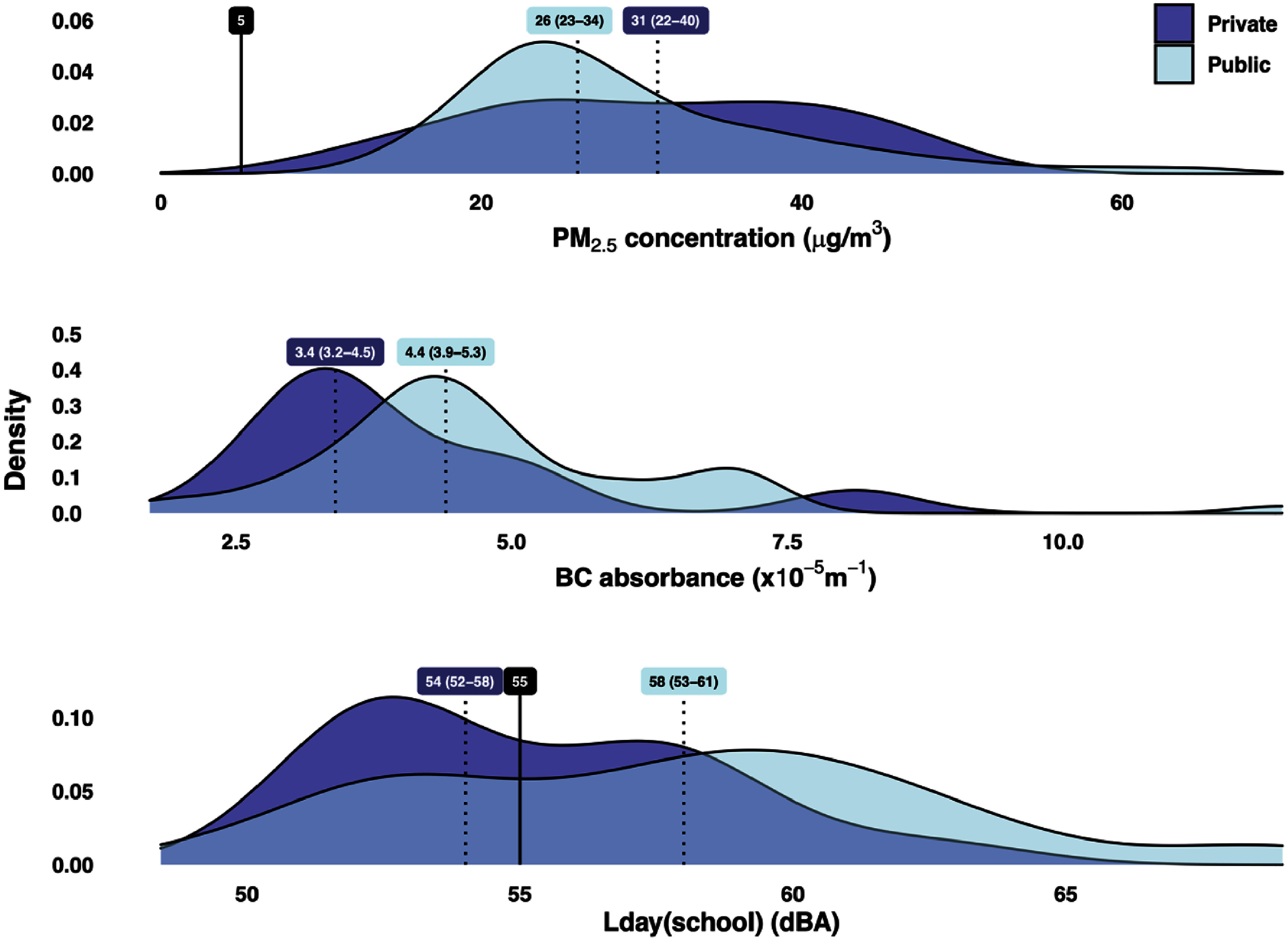
Distributions of annual equivalent PM_2.5_ and BC concentrations and environmental noise (*L*_day(school)_) levels across public and private schools. Dashed lines represent the median (IQR) pollutant levels while solid lines represent the WHO’s annual AQG for PM_2.5_ and the Ghana EPA’s standard for ambient noise at educational facilities.

The median (IQR) PM_2.5_ concentration across all 90 schools was 27 (22–36) *µ*g m^−3^, ranging from 11 *µ*g m^−3^ at a private school to 65 *µ*g m^−3^ at a public school (∼600% difference) (table 1). Annual equivalent PM_2.5_ levels in our study schools were 2–13 times higher than the WHO annual guideline of 5 *µ*g m^−3^ (figure 2). The concentrations in more than 55% of the schools were above even the second least stringent interim (IT-2) target of 25 *µ*g m^−3^. This held true even when PM_2.5_ concentrations were restricted to only the 7 am–3 pm school period on weekdays (figure 3). The median PM_2.5_ concentrations were higher in private schools, with 61% having levels above the median of all schools, compared to only 46% of public schools (table 1). Similar to PM_2.5_, BC levels varied widely by more than a factor of six between the least and most polluted schools. However, unlike PM_2.5_, BC levels were higher in public schools than in private schools (figure 4).

**Table 1.** Summary statistics of PM_2.5_ and BC concentrations, environmental noise levels and intermittency ratios (IR) across all schoolyards.

	Air pollutants		Noise metrics		
	PM_2.5_ (*µ*g m^−3^)	BC (1 × 10^−5^ m^−1^)	*L*_day(school)_ (dBA)	Leq_wk_ (dBA)	IR (%)
Mean (SD)	29.5 (10.4)	4.6 (1.7)	57.1 (4.6)	56.0 (4.5)	52.1 (10.5)
Median (IQR)	27.3 (22.3–35.8)	4.3 (3.6–5.1)	57.2 (52.9–60.1)	55.9 (52.6–58.3)	52.9 (46.2–58.1)
Range	11.2–64.8	1.7–12.0	48.4–69.0	48.5–65.2	12.9–72.6

The median (IQR) *L*_day(school)_ was 57 (53–60) dBA, with approximately 63% of schools having median levels exceeding 55 dBA, the Ghana EPA’s standard for daytime ambient noise at educational facilities. While only 48% of private schools exceeded this standard, 68% of public schools did so (figure 4). Also, 53% (IQR: 46%–58%) of the total noise was found to be created from distinct sound events that was louder than 3 dBA above the school and day-specific *L*_day(school)_ (table 1).

### 3.2. Disparities by spatial and socio-economic factors

By administrative districts, the median BC concentrations were higher at schools in the TMA (5.9 × 10^−5^ m^−1^) and AMA (4.7 × 10^−5^ m^−1^) when compared to schools in other districts (3.8 × 10^−5^ m^−1^). A similar trend was observed for environmental noise, as schools in the TMA and AMA were on average ∼2 dBA louder. While median PM_2.5_ concentrations were highest among schools in the TMA (28.5 *µ*g m^−3^), the levels were lower in the AMA (24.5 *µ*g m^−3^) when compared to all other GAMA districts (27.8 *µ*g m^−3^). There was a weak spatial correlation between concentrations of air pollutants and environmental noise, as high levels of PM_2.5_ and BC did not always coincide with high levels of noise in the schools, indicating the presence of both shared and distinct sources (figure S3).

In correlation analysis, distance (meters) of school location to the nearest major road was inversely associated with BC (*r* = − 0.29 [95% CI: −0.51, −0.05]) and *L*_day(school)_ (*r* = − 0.25 [95% CI: −0.44, −0.05]) but not with PM_2.5_ (*r* = 0.04 [95% CI: −0.17, 0.25]). Additionally, schools residing within 500 m of a major road had higher median levels of all pollutants when compared to those located beyond 500 m (figure S1). Schools located near a market (*n* = 16) had PM_2.5_ levels that were, on average, 9 *µ*g m^−3^ higher than the other schools. These schools also had median BC and *L*_day(school)_ levels that were 2.2 × 10^−5^ m^−1^ and 3 dBA higher, respectively. The median PM_2.5_ levels were 2 *µ*g m^−3^ lower in schools with paved yard surfaces than those with unpaved (dirt) surfaces. BC was also lower at schools with paved surfaces, but noise levels were unaffected. Furthermore, schoolyard NDVI score was inversely associated with BC (*r* = − 0.46 [95% CI: −0.64, −0.24]) and *L*_day(school)_ (*r* = − 0.23 [95% CI: −0.42, −0.02]), and schools with ‘bare soil’ had higher levels than those that had ‘sparse vegetation’ (figure S2).

**Figure 5.**
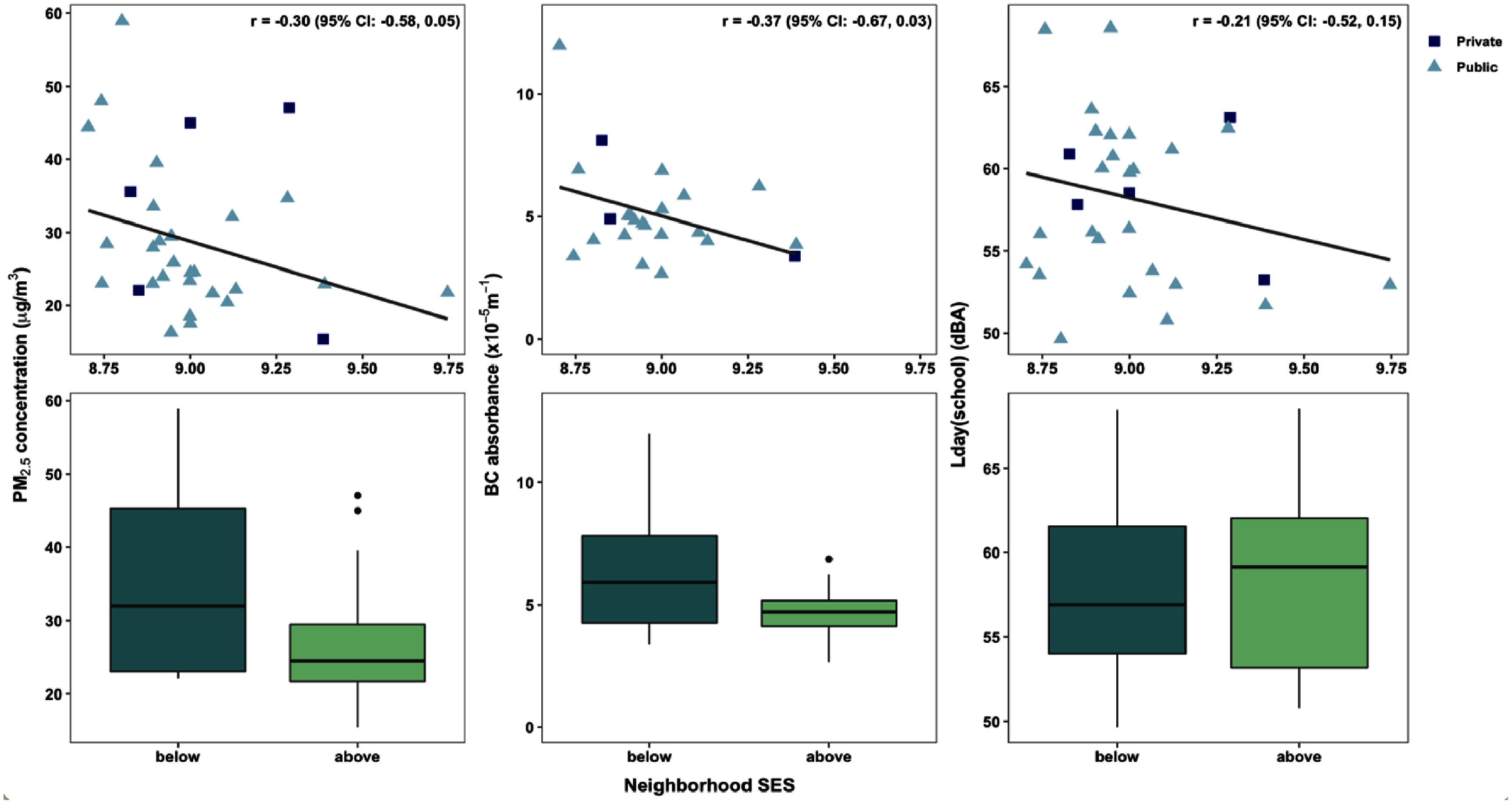
Association between school neighborhood SES and PM_2.5_, BC, and environmental noise among schools residing in the AMA (the main urban center of the GAMA). Scatterplots depict neighborhood SES on a continuous scale, while boxplots categorize schools as having neighborhood SES below or above the median SES index.

The PM_2.5_ concentrations in schools located in neighborhoods with SES indices below the median value were higher than in schools located in areas with SES indices above the median (29 vs 25 *µ*g m^−3^), though no differences were observed for BC or noise. Stronger disparities were observed when considering only those schools located in the AMA (*n* = 31), as levels of PM_2.5_ and BC were, on average, 7 *µ*g m^−3^ and 1.2 × 10^−5^ m^−1^ higher for schools below the median SES index. We also found inverse correlations between school neighborhood SES and PM_2.5_ (*r* = − 0.30 [95% CI: −0.58, 0.05]), BC (*r* = − 0.37 [95% CI: −0.67, 0.03]), and environmental noise levels (*r* = − 0.21 [95% CI: −0.52, 0.15]) among schools within the AMA (figure 5).

In multivariable analyses (table 2), schools located in the AMA and TMA had between 26%–55% higher average PM_2.5_ and BC concentrations than those outside of these districts. Furthermore, a one unit increase in schoolyard NDVI score was associated with ∼93% lower BC concentrations. Both PM_2.5_ and BC concentrations were ∼10% lower in schools with paved schoolyard surface than those with unpaved (dirt) yard surfaces, but the associations were weak. Public schools were ∼4 dBA (95% CI: 0.52, 7.08) noisier than private schools. In addition, market activity near a school was positively associated with the three pollutants, although not statistically significant. There were minimal associations with other spatial predictors (i.e. distance to major roads, distance to secondary roads, and SES Index) in our models.

**Table 2.** Associations of PM_2.5_, BC, and environmental noise with spatial and meteorological predictors.

	**ln(PM_2.5_)** *(Deviance = 84%)*	**ln(BC)** *(Deviance = 82%)*	**Leq_wk_** *(Deviance = 42%)*
**Predictor variables**	**Coefficient (95% CI)**		
District			
*Other GAMA districts*	—	—	—
*TMA*	0.52 (0.21, 0.83)	0.23 (−0.05, 0.51)	−3.05 (−6.13, 0.04)
*AMA*	0.26 (−0.06, 0.59)	0.43 (0.13, 0.74)	−0.05 (−3.09, 3.00)
Distance to major road	< −0.01 (< −0.01, <0.01)	< −0.01 (< −0.01, <0.01)	< −0.01 (< −0.01, <0.01)
Distance to secondary road	<-0.01 (< −0.01, <0.01)	< −0.01 (< −0.01, <0.01)	< 0.01 (< −0.01, <0.01)
Commercial activity			
*None*	—	—	—
*Market*	0.09 (−0.21, 0.41)	0.16 (−0.19, 0.52)	2.17 (−1.03, 5.38)
*Other*	−0.05 (−0.31, 0.19)	< −0.01 (−0.29, 0.29)	0.22 (−2.22, 2.67)
NDVI	1.80 (−0.62, 4.21)	−2.79 (−5.29, −2.92)	−21.44 (−48.90, 6.01)
SES Index	−0.20 (−0.50, 0.10)	0.04 (−0.25, 0.34)	−1.17 (−4.59, 2.26)
Schoolyard surface			
*Unpaved*	—	—	—
*Paved*	−0.10 (−0.28, 0.08)	−0.10 (−0.27 0.08)	2.78 (0.74, 4.83)
School type			
*Private*	—	—	—
*Public*	0.05 (−0.28, 0.39)	−0.03 (−0.34, 0.28)	3.80 (0.52, 7.08)
Season			
*Non-Harmattan*	—	—	—
*Harmattan*	−0.02 (−0.24, 0.19)	0.22 (0.03, 0.41)	0.17 (−2.16, 2.51)

**Predictor variables** ^a^	**Effective degrees of freedom (*p*-value)**		

Temperature^a^	5.05 (<0.001)	6.01 (0.001)	1.00 (0.472)
Relative Humidity^a^	1.92 (0.06)	1.00 (0.07)	1.00 (0.053)
Rain^a^	3.71 (<0.001)	3.33 (<0.001)	1.00 (0.059)

^a^ Thin plate regression splines were applied to temperature, relative humidity, and rain, as the relationship between these variables and the pollutants was non-linear.

### 3.3. Noise annoyance

**Figure 6.**
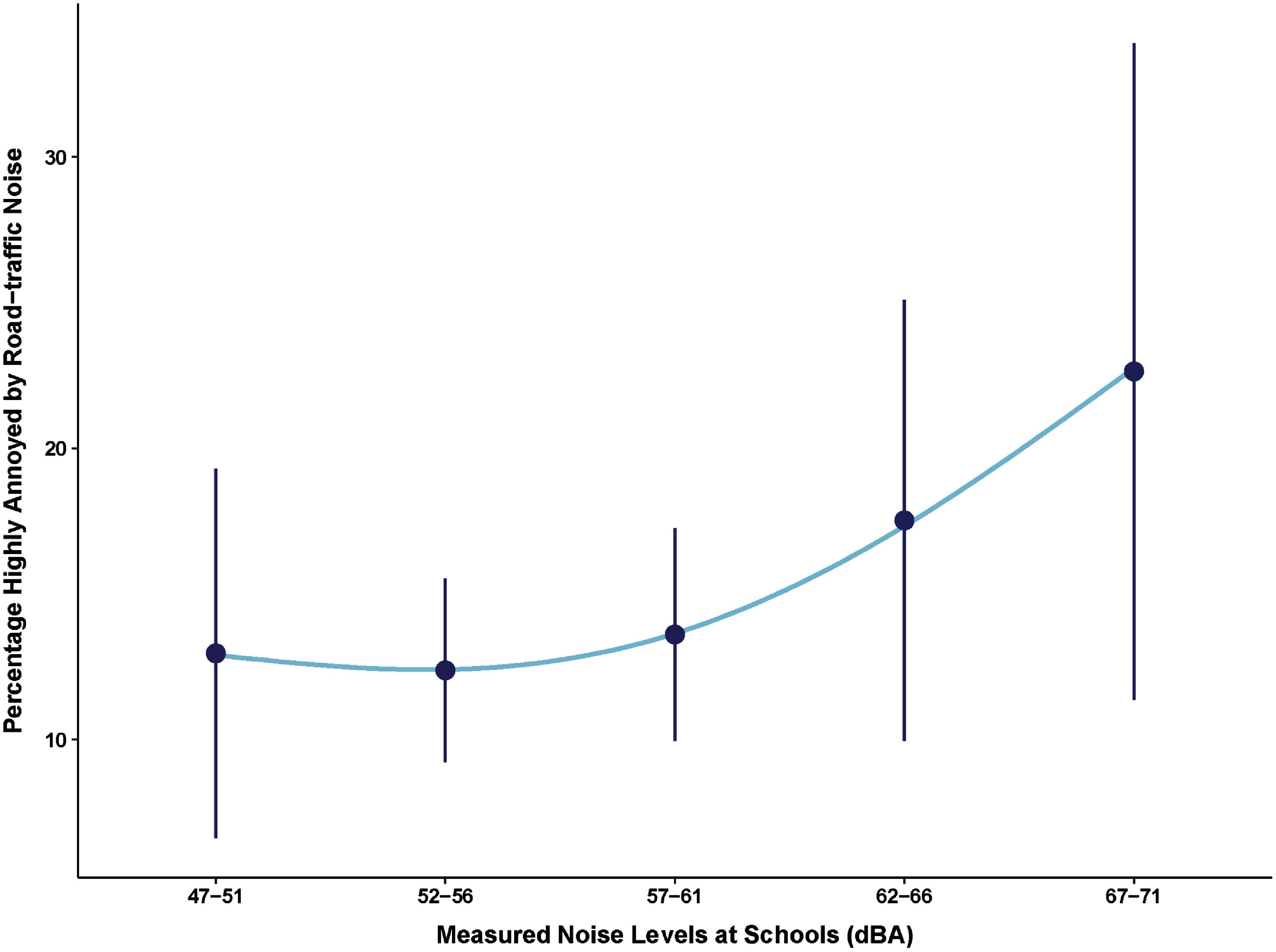
Scatterplot and quadratic regression of the relationship between *L*_day(school)_ and the calculated percentage of HA*_N_* for road-traffic noise at schools. The vertical lines represent the 95% CIs for each noise grouping.

The proportion of students highly annoyed (HA*_N_*) by noise from different sources near their schools ranged from as high as 60% due to neighbor noise, 14% due to road-traffic noise, and less than 10% due to noise from businesses/stores/churches, aircraft/planes, and industry/factories (table S1). Differences in responses were observed between districts, with children attending schools in the AMA and TMA generally reporting higher annoyance to noise than those residing in other districts (table S2). For example, approximately 72% of AMA and TMA respondents reported high annoyance to neighbor noise while only 51% of those in other districts did so. An annoyance exposure-response curve that examined the relationship between HA*_N_* to road-traffic noise and the measured school noise levels (*L*_day(school)_) showed that schools with higher levels of environmental noise had a higher percentage of students also reporting being HA to road-traffic noise (figure 6).

## 4. Discussion

To achieve universal primary education in SSA, more children are likely being enrolled in schools and new elementary schools are being built, primarily in rapidly sprawling cities where air and noise pollution present significant health concerns for children. Our study evaluated ambient PM_2.5_, BC, and noise pollution in 90 public and private primary schools in a major West African city. We found that every school (whether public or private) experienced high levels of air and noise pollution, with a large share of the students expressing a high level of noise annoyance. No school was close to meeting the international health-based guideline for ambient PM_2.5_, and roughly two-thirds of the schools were not meeting the local standard for noise at educational facilities. We also found higher BC and noise levels in public schools compared to private schools, and in schools located in the AMA and TMA when compared to other districts in the GAMA. We further observed higher levels of all three pollutants in schools residing in lower-SES neighborhoods in the AMA.

Like our study, recent work documented mean PM_2.5_ concentrations several times above the WHO AQG [47] in outdoor playgrounds of elementary schools in Kigali, Rwanda. Furthermore, PM_2.5_ concentrations in our study were higher than those reported in elementary schools in the UK (mean: 9.1 *µ*g m^−3^) [48], US (mean: 6.5 *µ*g m^−3^) [49], Italy (mean: 14.2 *µ*g m^−3^) [50] and South Africa (mean: 20.7 *µ*g m^−3^) [51], but lower than those observed at schools in China (mean: 63 *µ*g m^−3^) [52] and India (mean:135 *µ*g m^−3^) [53]. Environmental noise in schools in Accra was on average 10 dBA lower than those reported at four schools in the Greater Kumasi Metropolis (Ashanti Region), the second largest city in Ghana [54]. However, we observed higher average noise levels compared to schools in European countries such as those in Spain (53.5 dBA) [55], Germany (50.6 dBA) [56] and France (51.5 dBA) [57].

We found associations between the measured pollutants and several spatial factors, including distance to major roads, vegetation/greenness of the schoolyard, schoolyard surface, and commercial activity near the school. These factors have also been identified in previous studies in Accra [45, 46]. Similar to ours, studies from developed countries have also documented the influence of green spaces surrounding schools on pollutant concentrations [27, 58]. In fact, a recent review of the literature showed that installing green infrastructure as a physical barrier can reduce BC concentrations at schools by up to 63% [59]. This suggests that enhancing greenness at schools within the GAMA may be an effective strategy for reducing children’s exposure to both air and noise pollution, particularly at schools with the lowest NDVI scores. Furthermore, our finding of inverse associations between neighborhood SES and pollutant levels are consistent with a much wider body of literature on childhood SES and disparities in pollution exposures, emphasizing the need to identify and resolve these exposure disparities in Accra [48, 60, 61]. Finally, the percentage of children who reported being HA to road traffic noise was comparable to findings from previously published meta-analyses aggregating results from studies amongst adults globally [62]. These findings underscore the importance of reducing road-traffic noise by encouraging local government in Accra to identify and target predominant noise sources (e.g. engines, honking, tire contact, etc.)

Unlike BC and noise, the relationship between PM_2.5_ and the spatial factors was less apparent. As noted in a previous study [37], PM_2.5_ pollution in Accra has become more uniform across communities and not as much affected by local sources as was documented in the past. This could explain the relatively weak association with the spatial factors. In contrast, BC and environmental noise in the schools are better predicted by local pollution sources [63, 64]. While the associations between neighborhood SES and pollutant levels were weak in our multivariate models, the link was stronger in the AMA, the most urbanized part of the GAMA. This could be because SES was shown to vary more widely in the inner-city core than in the neighboring communities [65].

### 4.1. Strengths and limitations

Our study has several innovations and strengths. To the best of our knowledge, this is the largest air and noise pollution study in schools in a major SSA city, a region where cities are experiencing high levels of environmental pollution but lack measurement data to motivate policy actions. Our design approach allowed for the assessment of multiple air pollutants and indicators of environmental noise pollution in schools across the entire metropolis with varied land use and population features. Additionally, for the first time in SSA, we captured the perception of schoolchildren in terms of their annoyance to environmental noise at schools. While policy interventions are primarily implemented through public schools, we also included a representative sample of private schools for comparison, as they have become a significant part of the educational system in SSA and highlight major inequalities in pollution exposures between fee and non-fee paying schools. With the large sample size, we have identified school- and community-level factors that can be a focus of targeted policy interventions to reduce exposures at school environments.

Our study also has some limitations. Due to logistical difficulties, including a limited number of monitors, the large number of schools involved, and the large study area, we could not conduct measurements in all our study schools simultaneously. However, to allow for comparisons across schools, we adjusted for the effect of season using concurrent measurements at the ten fixed sites located across the city, an approach which has been used in previous studies. For the same reason, we could not conduct in-classroom and personal exposure measurements in the children, which would provide additional information about schoolchildren exposures in Accra. Nevertheless, we captured data on some of the school-level factors (e.g. schoolyard surface, distance of school location to main road) that were found to be determinants of personal exposure among schoolchildren in Accra [28]. Furthermore, the standardized noise annoyance questions used in this research have only been validated for use at homes and among adult populations [42], and not specifically among schoolchildren. However, the proportion of children identified as HA by road traffic noise was similar to findings from previously published studies [62]. Nonetheless, the results should be interpreted with caution. Finally, our measure of neighborhood SES included the 2010 census and 2012 GLSS data. While these were the most recent data available, sociodemographic dynamics have likely shifted since then, with many areas becoming more developed and higher income. As a result, the relationship between SES and pollutant concentrations may be underestimated, as these areas may now experience lower pollution levels than reflected by older SES data.

## 5. Conclusion

Our findings show that children in elementary schools in Accra are learning in polluted air and annoyingly noisy environments, raising concerns about their physical, cognitive, and social well-being. No school (private or public) is close to meeting the international health-based guidelines or local standards for ambient air and environmental noise pollution. Children in public schools, and those attending schools located in the more urbanized areas, are at higher risk of exposure. As SSA works towards its goal of universal primary education, our data highlight an urgent need for polices aimed at reducing children’s exposures to air and noise pollution in urban schools. Effective actions could include paving the schoolyards using brick or concrete-based pavement tiles and introducing green spaces on school grounds. Additionally, the planning and construction of new schools should consider proximity to pollution sources. While public schools may warrant particular attention due to existing disparities, broader policy discussions are needed to ensure all GAMA students, regardless of school type, have equitable access to clean and quiet learning environments. Mitigating these exposures is an important step toward supporting the health and academic success of future generations.

## Data Availability

Environmental measurement data is available through the open-source platform, Zenodo. Those interested in accessing the data should direct any questions to Raphael E Arku [rarku@umass.edu]. Supplementary Information available at https://doi.org/10.1088/2752-5309/ae27eb/data1
